# Supplementary material for: Reader domain specificity and lysine demethylase-4 family function
Source: Nat Commun. 2016 Nov 14;7:13387. doi: 10.1038/ncomms13387 (PMC5114558; doi:10.1038/ncomms13387)
Supplement: Supplementary Information — Supplementary Figures 1-10 and Supplementary Tables 1-2. [file ncomms13387-s1.pdf]

Supplementary information

Supplementary Figure 1.

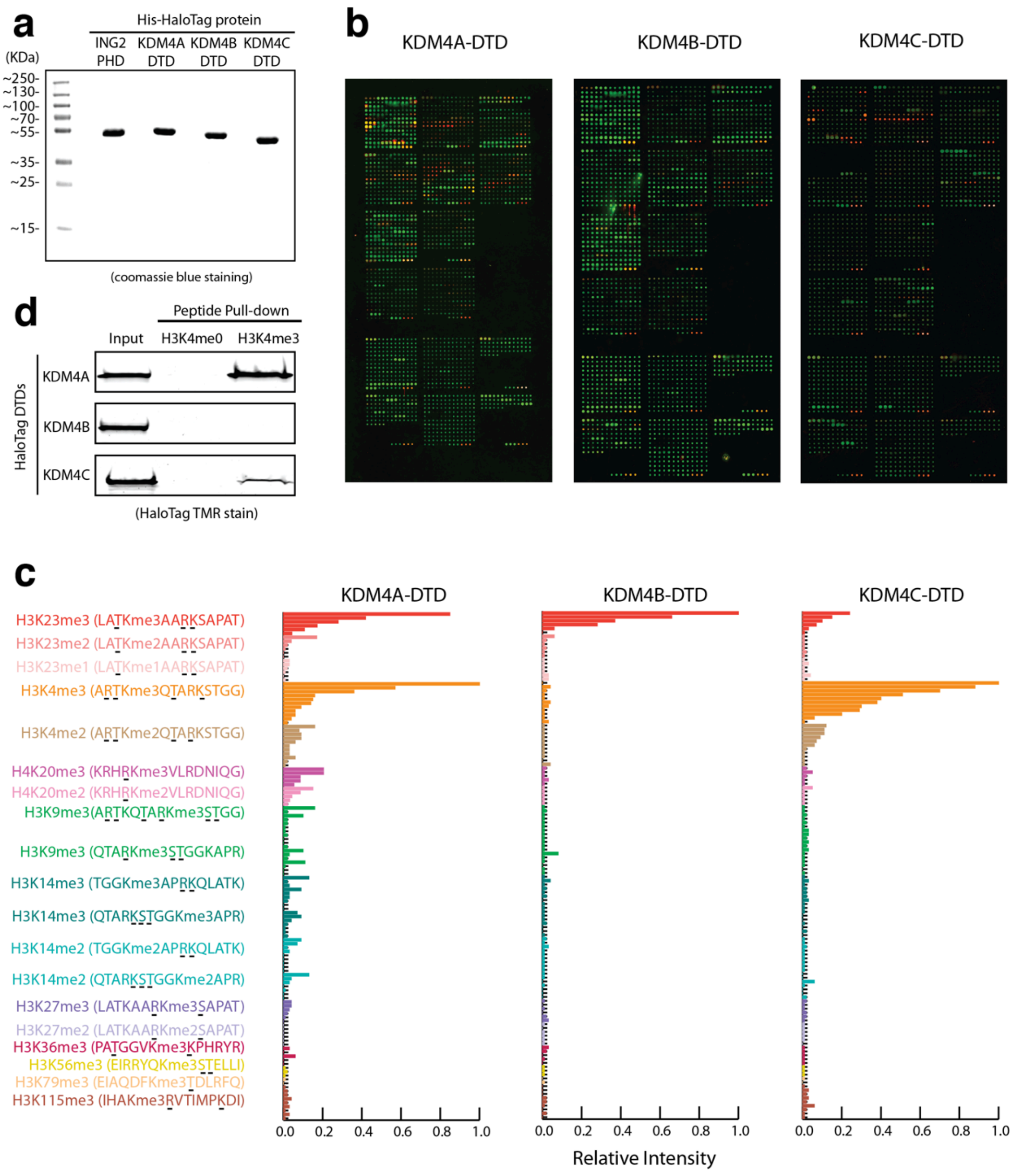

Supplementary Figure 1. Different histone binding specificities of human KDM4A-C double tudor domains.

(a) Purified His-HaloTagged KDM4A-C DTDs were resolved on 12% SDS-PAGE and stained with coomassie blue.

(b-c) Systematic profiling of histone binding preferences of KDM4A-C DTDs on histone peptide microarray. (b) Representative images are shown for KDM4A-C DTDs. Red signals (635nm channel) correlate with binding events and green signals (532 nm channel) represent printing tracer dye. (c) Relative intensity for selective peptide species from KDM4-DTD probed peptide microarray. Relative signal intensity is calculated by normalizing each mean signal intensity at 635 nm of triplicate spots to the highest signal on individual sub-array, after subtracting background signals (derived from empty spots) for all spots. Peptide species containing the same PTMs are grouped together with indicated peptide sequence. Underline in each sequence represents permutated residues in peptides that have additional PTMs.

(d) Decreased H3K4me3 binding of KDM4B-DTD compared with KDM4A and KDM4C DTDs by biotinylated histone peptide pull-down.

Supplementary Figure 2.

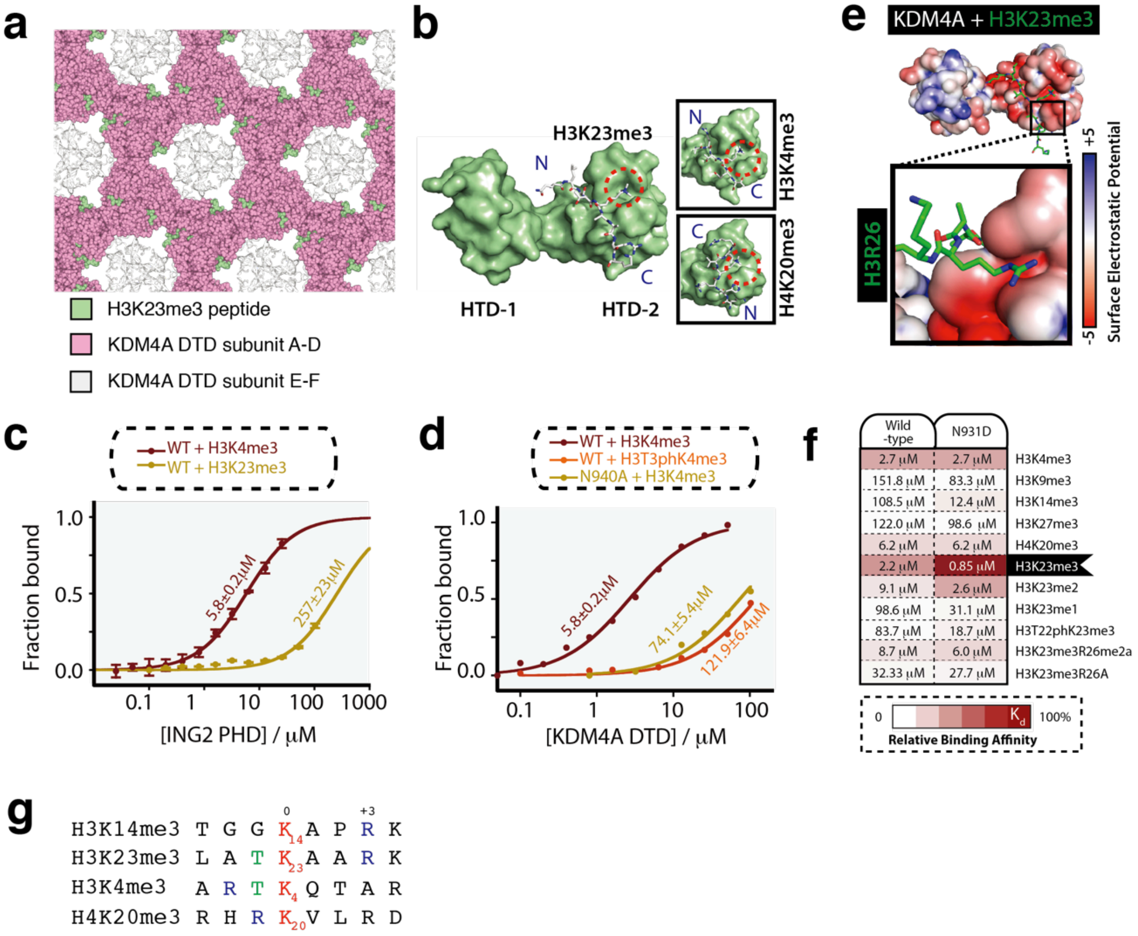

Supplementary Figure 2. Co-crystal structure of KDM4A-DTD with meiosis-specific PTM H3K23me3.

(a) Packing view of six subunits in the KDM4A-H3K23me3 structure (PDB ID: 5D6Y). Green: H3K23me3 peptide; pink: KDM4A DTD subunits A-D; gray: partially disordered KDM4A DTD subunits E-F.

(b) Comparison of histone peptide orientation in the KDM4A-DTD co-crystal structures (H3K4me3 - PDB ID: 2GFA, H4K20me3 - PDB ID: 2QQS and H3K23me3 - PDB ID: 5D6Y).

(c) H3K4me3-reader ING2-PHD does not interact with H3K23me3 as determined by fluorescence polarization.

(d) KDM4A-N940 and H3T22 interaction pair is important for H3K4me3 binding.

(e) Surface electronic potential of H3K23me3 binding by KDM4A-DTD. The zoomed in box highlights H3R26 binding site.

(f) KDM4A-DTD N931D specifically increases H3K23me3 binding. Labeled in each box is the K_d value determined by fluorescence polarization. Shade of red indicates the relative binding affinity normalized to the tightest binding (K_d = 0.85 μM).

(g) Sequence of surrounding residues for H3K14, H3K23, H3K4 and H4K20.

Supplementary Figure 3.

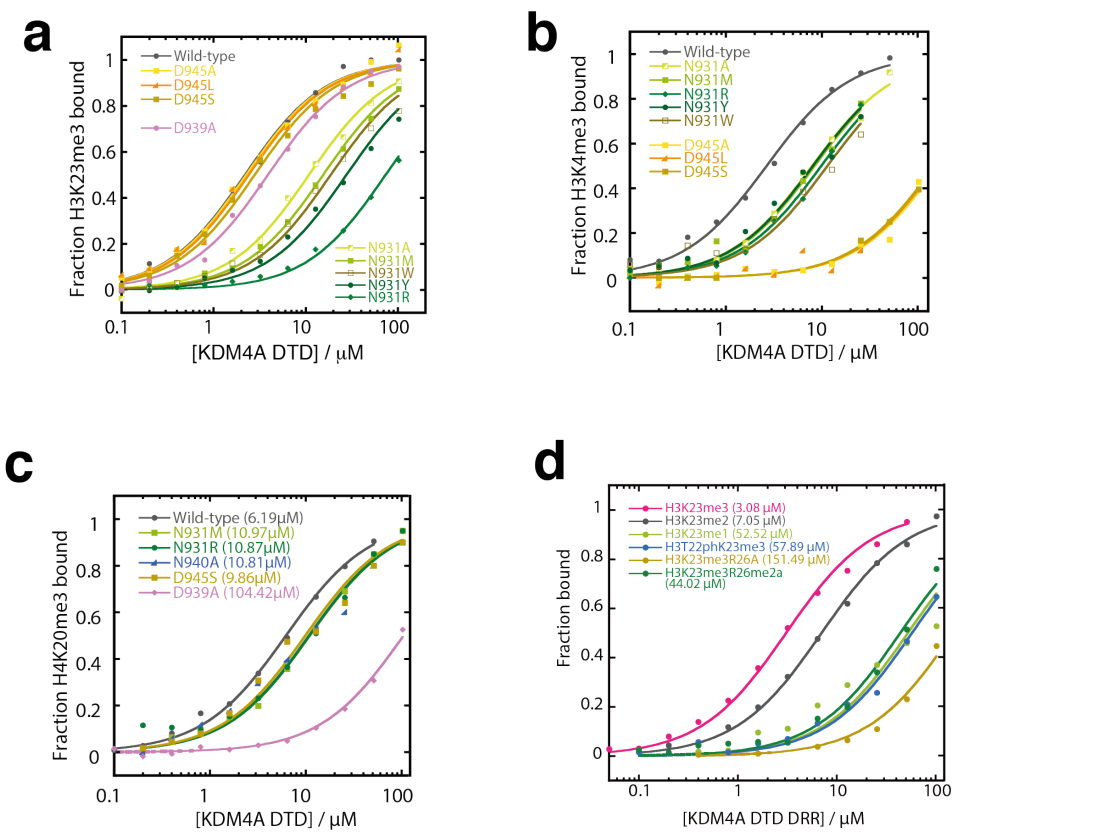

<u>Supplementary Figure 3. H3K23me3 recognition is mediated by unique side-chain interactions.</u>

(a-c) Different binding modes of H3K4me3, H3K23me3 and H4K20me3 can be separated by specific sets of mutations at D945, N931 and D939 of KDM4A-DTD.

(d) Sequence specificity and PTM specificity of KDM4A-DTD DRR mutant. K_d values are measured by fluorescence polarization assays and labeled for each binding in parenthesis.

Supplementary Figure 4.

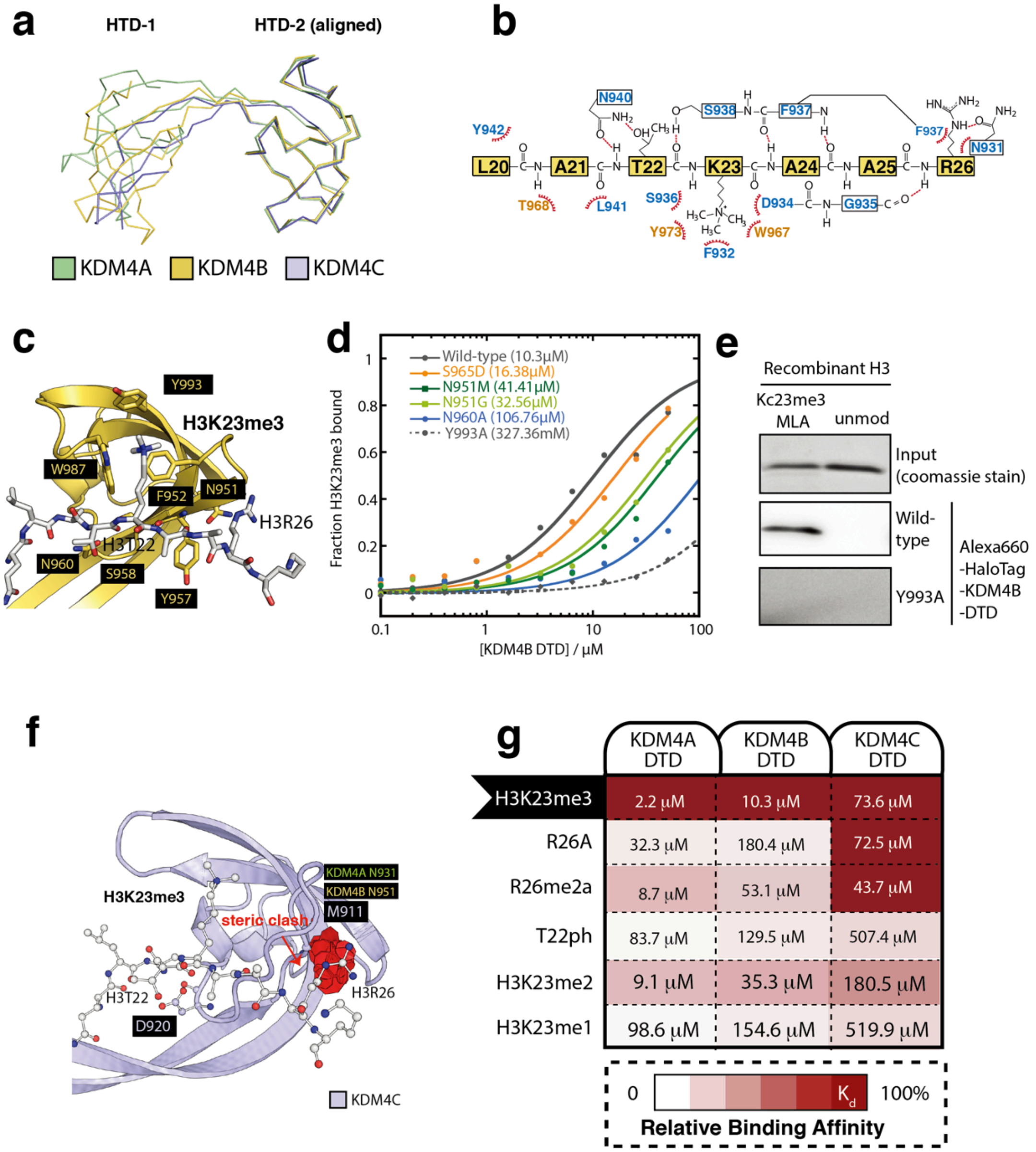

Supplementary Figure 4. KDM4B-DTD is an H3K23me3-specific reader.

(a) Ribbon representation of KDM4A-C DTD overall structures (KDM4A: 2GFA, KDM4B: 4UC4, KDM4C: 2XDP) aligned by HTD-2 domains.

(b-c) Interaction network in the modeled KDM4B-H3K23me3 structure.

(d) H3K23me3 binding of KDM4B-DTD mutants (measured by fluorescence polarization).

(e) KDM4B-DTD wild-type but not Y993A mutant recognizes H3Kc23me3 methyl-lysine analog (MLA) in a far western setting. Recombinant H3 without any modification was included as control.

(f) Clash observed between H3R26 and M911 in modeled KDM4C-H3K23me3 structure.

(g) Effects of H3K23 methylation state and secondary modifications on H3T22/R26 for H3K23me3 binding with KDM4B-DTD and KDM4C-DTD. KDM4B-DTD display similar molecular network as KDM4A-DTD for H3K23me3 binding, whereas KDM4C lacks H3R26 interaction.

Supplementary Figure 5.

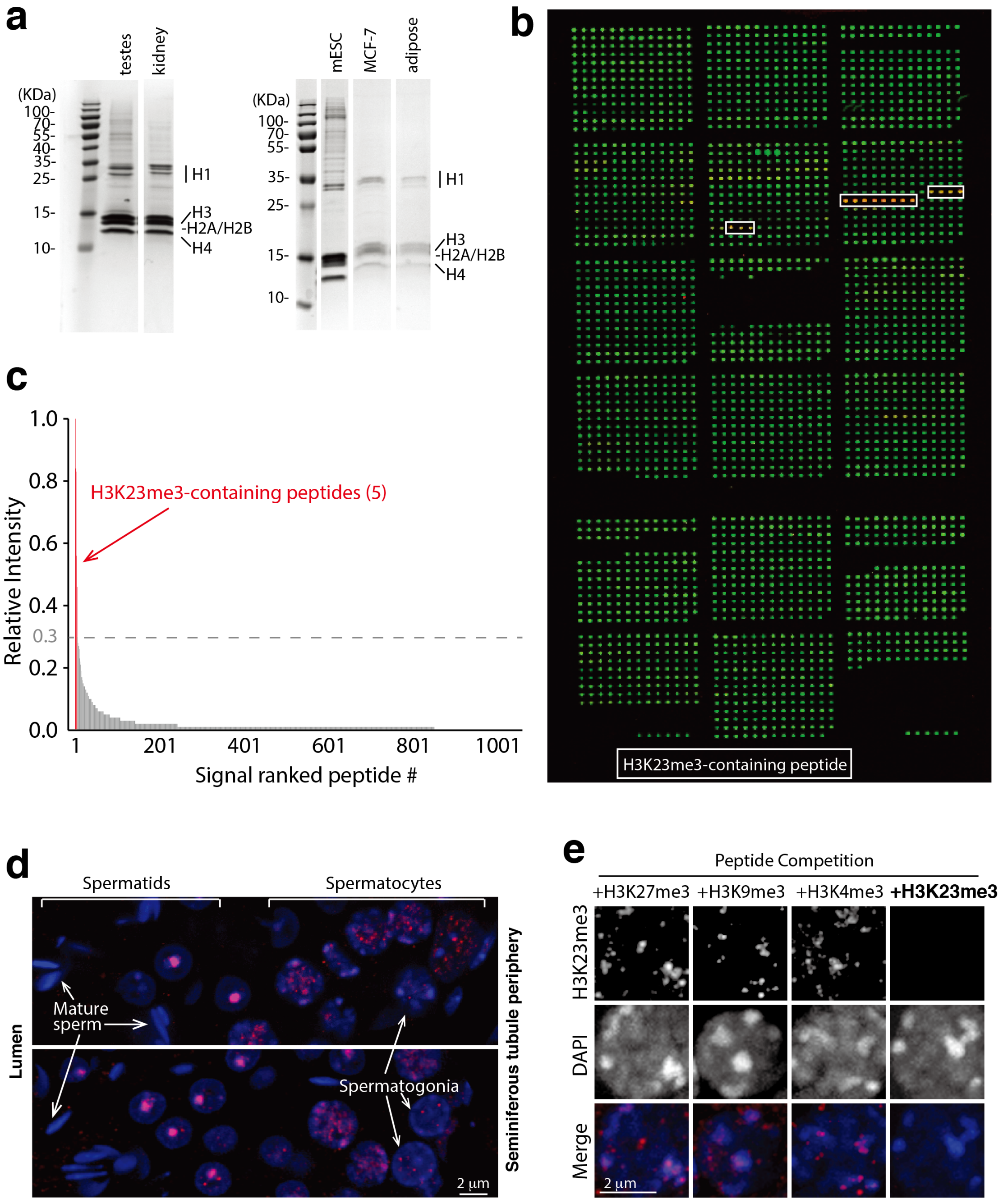

Supplementary Figure 5. Profiling H3K23me3 with H3K23me3 antibody.

(a) Histone preparation from different mammalian cell lines and mouse tissues by acid extraction.

(b-c) Systematic profiling of H3K23me3 antibody specificity on histone peptide microarray. (b) Representative images are shown for H3K23me3 antibody binding. Red signals (635nm channel) correlate with binding events and green signals (532 nm channel) represent printing tracer dye. White boxes denote H3K23me3-containing peptides. (c) Ranked signal distribution for all peptide species in H3K23me3-binding peptide microarray. Relative signal intensity (y-axis) is calculated by normalizing each mean signal intensity at 635 nm of triplicate spots to the highest signal on individual sub-array, after subtracting background signals (derived from empty spots) for all spots. The dash line represents the cut-off at 0.3. The top five highest binding peptide species are H3K23me3-containing peptides.

(d) Representative immunofluorescence images of mouse testes. H3K23me3 antibody (red) overlapped with DAPI stain (blue).

(e) Immunofluorescence with H3K23me3 antibody pre-incubated with competing peptides (H3K27me3, H3K9me3, H3K4me3 and H3K23me3). Only H3K23me3 peptide is able to compete off H3K23me3 antibody binding, demonstrating the specificity of this antibody.

Supplementary Figure 6.

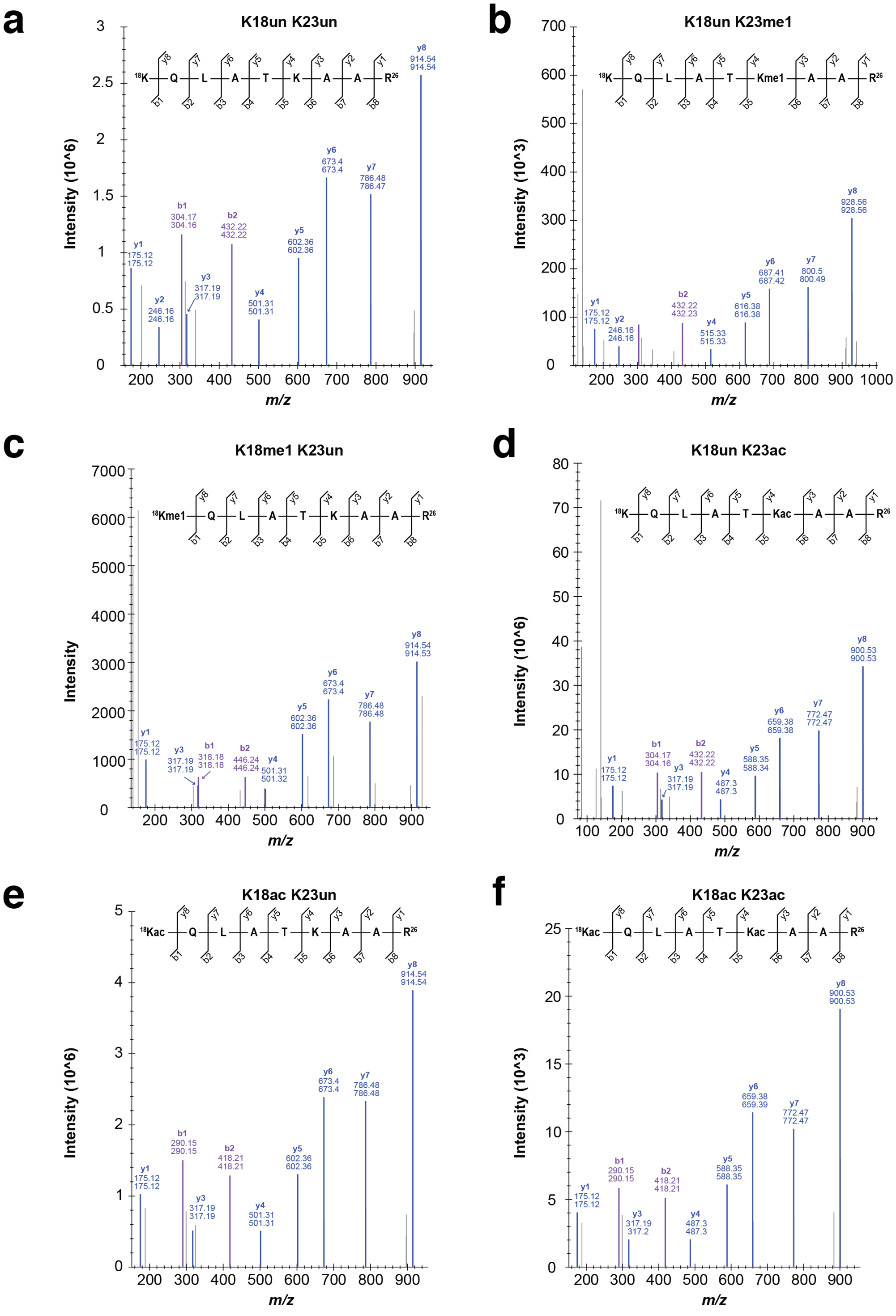

Supplementary Figure 6. MS2 spectra for 6 permutations of the histone H3 K18-R26 peptide.

MS2 spectra for 6 permutations of the histone H3 K18-R26 peptide (KQLATKAAR): (A) K18unK23un, (B) K18un K23me1, (C) K18me1 K23un, (D) K18un K23ac, (E) K18ac K23un, and (F) K18ac K23ac. The relevant peptide is denoted above each sub-figure. Purple denotes b-ions and blue denotes y-ions. The expected m/z based on the spectral library is listed at the top of each transition, and the observed m/z is listed directly below it.

Supplementary Figure 7.

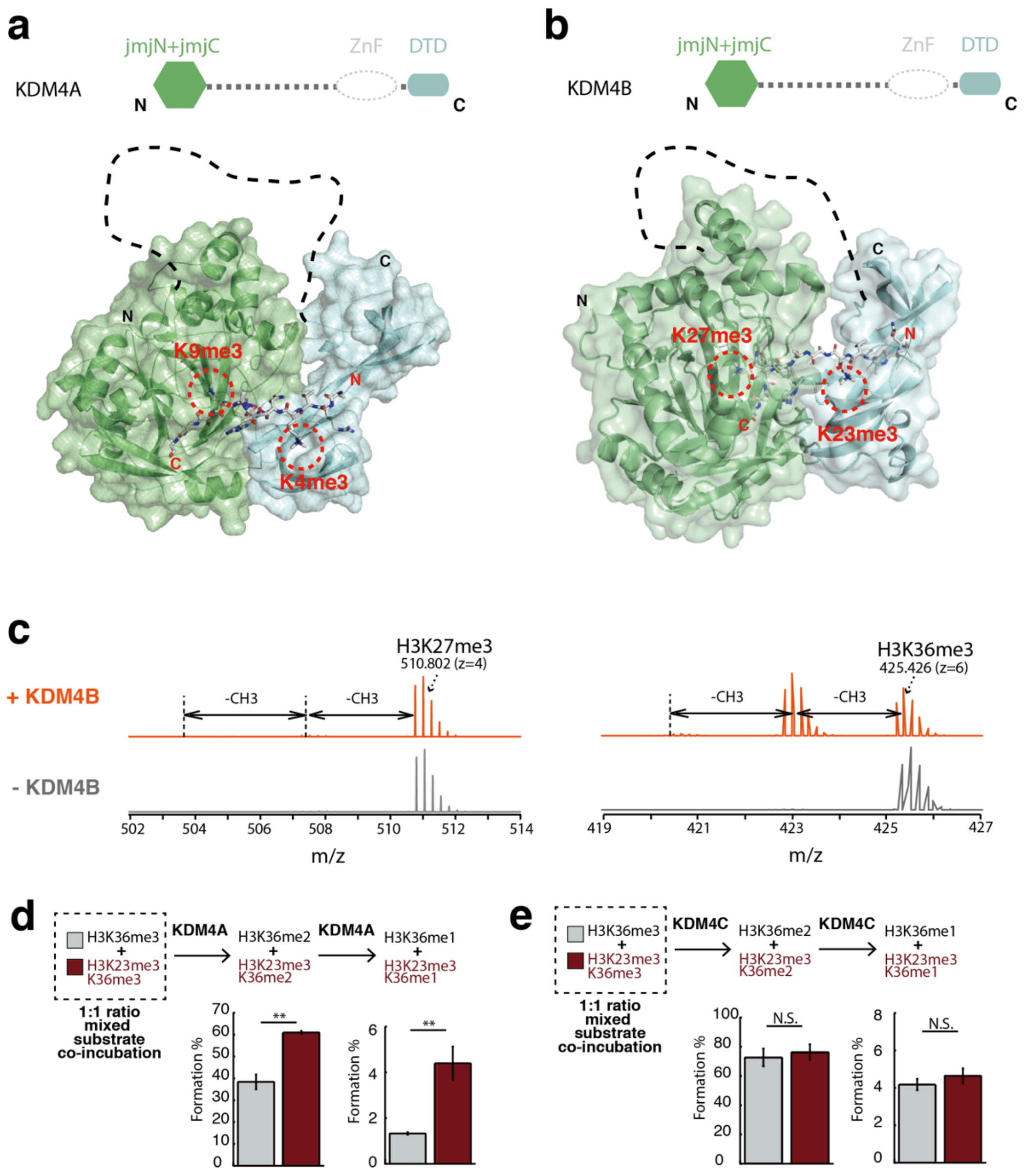

Supplementary Figure 7. H3K23me3-mediated demethylation by KDM4B.

(a-b) Structural models of simultaneous coordination by catalytic domain and reader domain in KDM4A/B. (a) H3(1-13)K4me3K9me3 peptide was modeled with KDM4A. (b) H3(19-28)K23me3K27me3 peptide is modeled with KDM4B. N-terminal catalytic domain is shaded in green color and C-terminal double tudor domain is shaded in teal color. The middle portion that is missing from the model is represented by the dashed line.

(c) Mass spectrometry analysis of demethylation reaction by KDM4B full-length protein. $H3_{(17\text{-}34)}$K27me3 (c) or $H3_{(20\text{-}42)}$K36me3 (d) peptides were used as substrates. Negative controls without KDM4B enzyme were shown side-by-side. The shown spectrums of only one charge state at one time point (H3K27me3 peptides: +4 charge state, 120 minutes; H3K36me3 peptides: +6 charge state, 30 minutes) display representative trends of all three charge states at three time points for each peptide.

(d-e) KDM4A but not KDM4C preferentially demethylates dually modified peptide in 1:1 (5 μM: 5 μM) ratio mixture of H3K36me3 and H3K23me3K36me3 substrate. Site and level of demethylation was quantified by mass spectrometry. Errors represent standard deviation (S.D.) from three charge states of the same peptide.

Supplementary Fig. 8

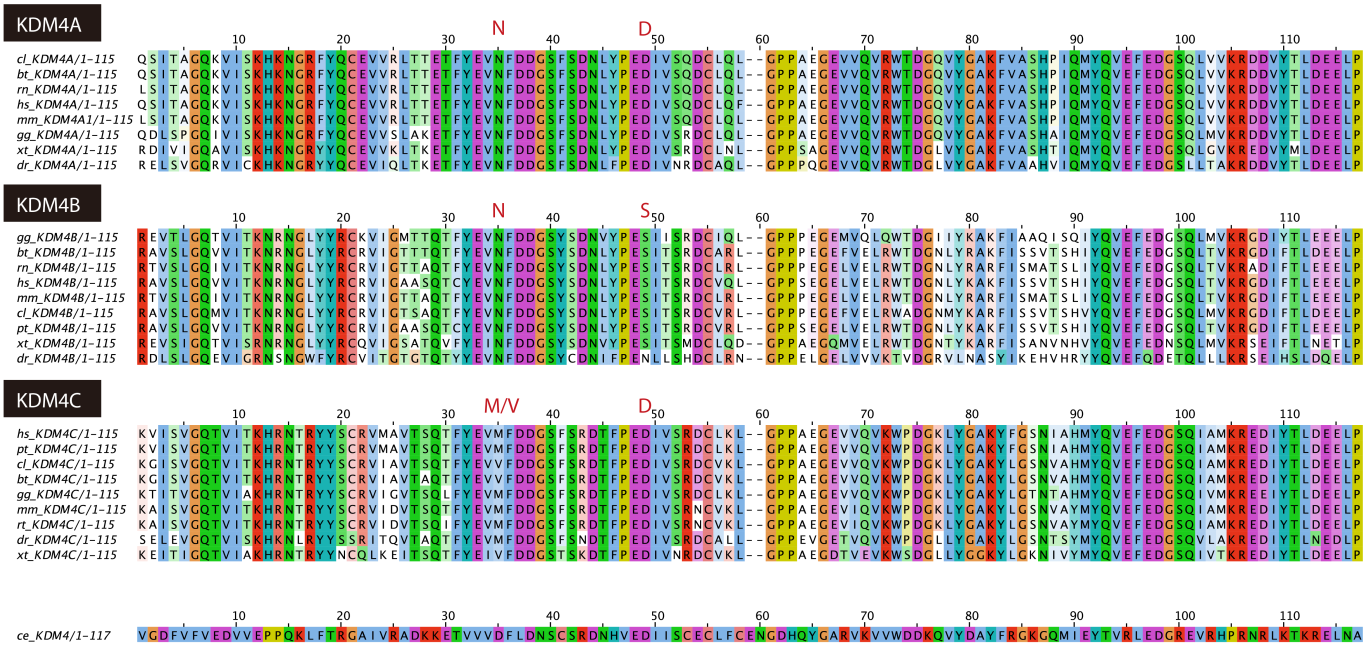

Supplementary Figure 8. Sequence alignment of double tudor domains from KDM4 members across multiple species.

Sequence alignment is generated by Jalview 1.0 (bt = *Bos Taurus*, ce = *Caenorhabditis elegans*, cl = *Canis lupus familiaris*, dr = *Danir rerio*, gg = *Gallus gallus*, hs = *Homo sapiens*, mm = *Mus musculus*, pt = *Pan troglodytes*, rn = *Rattus norvegicus*, xt = *Xenopus tropicalis*).

Supplementary Fig. 9

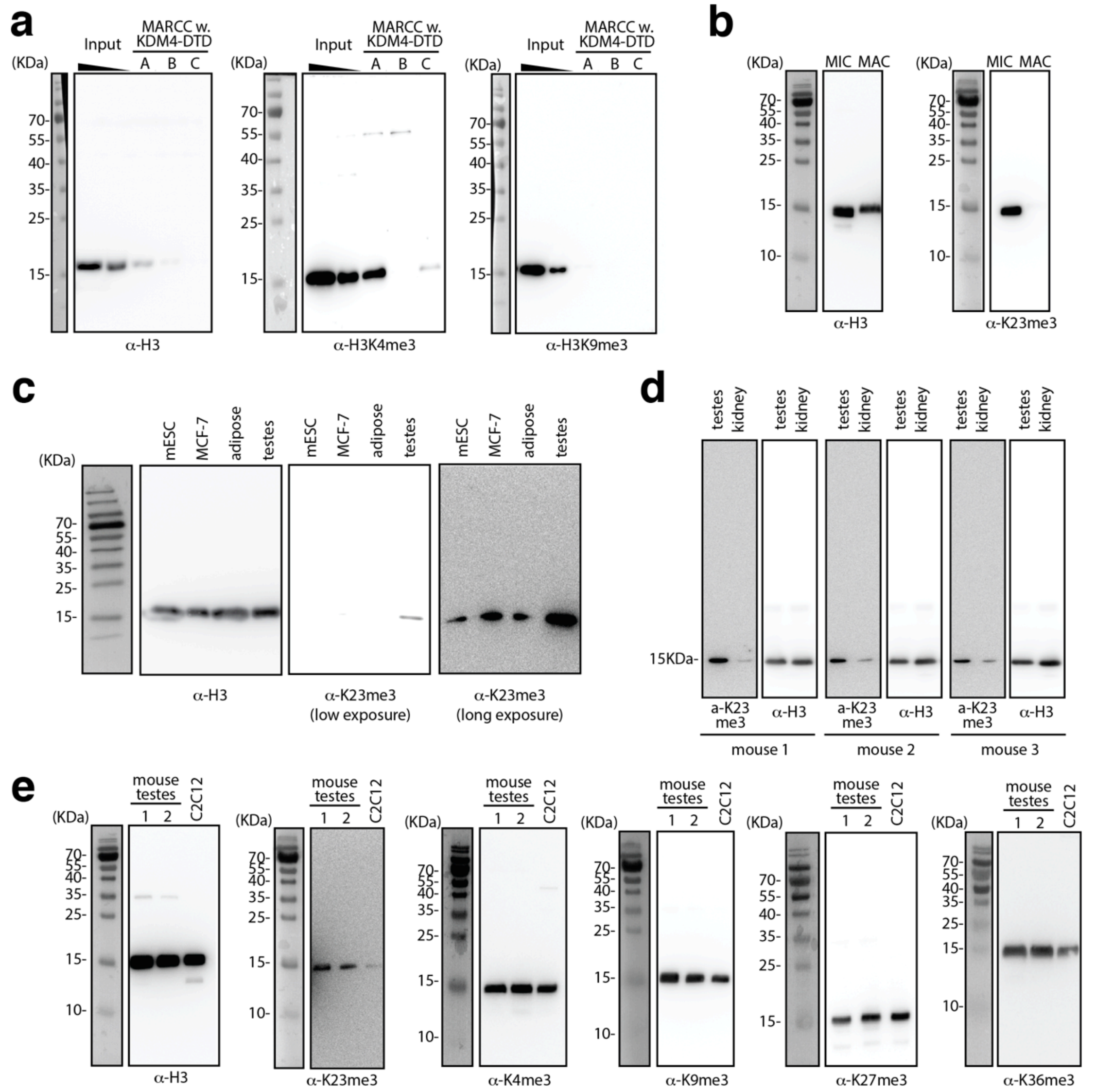

<u>**Supplementary Figure 9. Full-size images for western blots in Fig. 4c (a), Fig. 5a (b-d) and Fig. 5b (e).**</u>

Supplementary Fig. 10

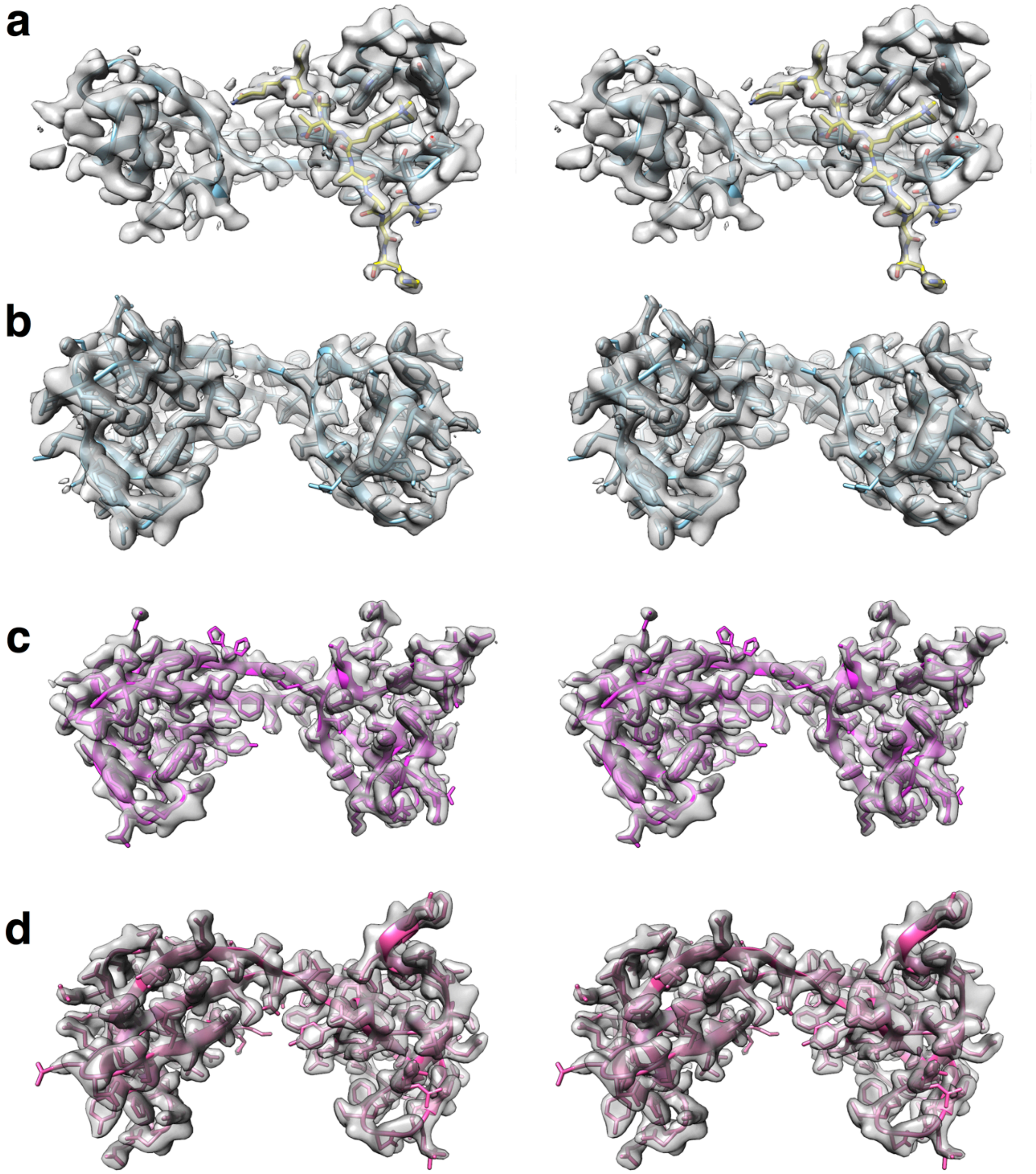

Supplementary Figure 10. Stereoview of the electron density map for all crystallographic structures (2mFo-DFc map, contoured at 1.2σ).

(a) KDM4A-H3K23me3 structure (PDB ID: 5D6Y). Yellow: H3K23me3 peptide; cyan: KDM4A-DTD subunits. Side chains of the H3K23me3 peptide and key residues of KDM4A-DTD are displayed.

(b) KDM4B-DTD structure (PDB ID: 4UC4). All side chains are displayed as sticks.

(c) and (d) KDM4A-DTD structures (PDB ID 5D6W and 5D6X). All side chains are displayed as sticks.

Supplementary Table 1. List of soluble peptides used in the study.

Name	Sequence	Use	Source
H3K4me3	ARTK(Me3)QTARKSTGGK-(5-FAM)	FP	In-house
H3K4me3+T3ph	ART(Ph)K(Me3)QTARKSTGGK-(5-FAM)	FP	In-house
H3K4me3	ARTK(Me3)QTARKSTE(PEGbiotin)W	Pull-down	In-house
H3unmod	ARTK(Me3)QTARKSTE(PEGbiotin)W	Pull-down	In-house
H3K23me1	(5-FAM)-RKQLATK(Me1)AARKSAPAT	FP	In-house
H3K23me2	(5-FAM)-RKQLATK(Me2)AARKSAPAT	FP	In-house
H3K23me3	(5-FAM)-RKQLATK(Me3)AARKSAPAT	FP	In-house
H3K23me3	QLATK(Me3)AARKS	Co-crystal	Peptide 2.0
H3K23me3+T22ph	(5-FAM)-RKQLAT(Ph)K(Me3)AARKSAPAT	FP	In-house
H3K23me3+R26A	(5-FAM)-RKQLATK(Me3)AAAKSAPAT	FP	In-house
H3K23me3+R26me2a	(5-FAM)-RKQLATK(Me2)AAR(Me2a)KSAPAT	FP	In-house
H4K20me3	(5-FAM)-AKRHRK(Me3)VLRDN	FP	In-house
H3K9me3	ARTKQTARK(Me3)STGGKK-(5-FAM)	FP	In-house
H3K14me3	(5-FAM)-ARKSTGGK(Me3)APRKQLATK	FP	In-house
H3K27me3	(5-FAM)-RKQLATKAARK(Me3)SAPAT	FP	In-house
H3K27me3	RKQLATKAARK(Me3)SAPATGGW	Demethylation assay	Peptide 2.0
H3K23me3+K27me3	RKQLATK(Me3)AARK(Me3)SAPATGGW	Demethylation assay	Peptide 2.0
H3K36me3	(Ac)-LATKAARKSAPATGGVK(Me3)KPHRYR-(NH2)	Demethylation assay	Tufts University Core Facility
H3K23me3+K36me3	(Ac)-LATK(Me3)AARKSAPATGGVK(Me3)KPHRYR-(NH2)	Demethylation assay	Tufts University Core Facility

Supplementary Table 2. Radius of gyration for KDM4A-C DTDs.

Name	PDB ID	MW (Da)[a]	Elution volume (ml)	R_g[b] (chain A/B)
KDM4A DTD	2QQR, 2GF7, 5D6W, 5D6X	13,715	90	17.0 / 16.5
KDM4B DTD	4UC4	13,461	92	16.8 / 18.5
KDM4C DTD	2XDP	14,416	90	17.6
Ribonuclease A	7RSA	13,782	96	14.5
Carbonic Anhydrase	1CA2	29,223	89	17.4

[a]Molecule weight of protein

[b]Radius of gyration (R_g) describes the overall spread of the molecule and is defined as the root mean square distance of the collection of atoms from their common center of gravity, and is calculated as $R_g^2 = \sum_i (m_i(\vec{r_i} - \vec{r_c})^2) / \sum_i m_i$, where r_c is the center of mass, m_i is the mass of the i-th atom and r_i shows its coordinates.
